# Development and Preliminary Testing of a Framework to Evaluate Patients' Experiences of the Fundamentals of Care: A Secondary Analysis of Three Stroke Survivor Narratives

**DOI:** 10.1155/2013/572437

**Published:** 2013-06-20

**Authors:** Alison L. Kitson, Åsa Muntlin Athlin

**Affiliations:** ^1^School of Nursing, Faculty of Health Sciences, University of Adelaide, Level 3, Eleanor Harrald Building, Adelaide, SA 5005, Australia; ^2^Centre for Evidence Based Practice South Australia, School of Nursing, Faculty of Health Sciences, The University of Adelaide, Adelaide, SA 5005, Australia; ^3^Green Templeton College, University of Oxford, 43 Woodstock Road, Oxford OX2 6HG, UK; ^4^Department of Medical Sciences, Uppsala University, Uppsala University Hospital, 751 85 Uppsala, Sweden; ^5^Department of Public Health and Caring Sciences, Uppsala University, Box 564, 751 22 Uppsala, Sweden; ^6^Department of Emergency Care, Uppsala University Hospital, 751 85 Uppsala, Sweden

## Abstract

*Aim*. To develop and test a framework describing the interrelationship of three key dimensions (physical, psychosocial, and relational) in the provision of the fundamentals of care to patients. *Background*. There are few conceptual frameworks to help healthcare staff, particularly nurses, know how to provide direct care around fundamental needs such as eating, drinking, and going to the toilet. *Design*. Deductive development of a conceptual framework and qualitative analysis of secondary interview data. *Method*. Framework development followed by a secondary in-depth analysis of primary narrative interview data from three stroke survivors. *Results*. Using the physical, psychosocial and relational dimensions to develop a conceptual framework, it was possible to identify a number of “archetypes” or scenarios that could explain stroke survivors' positive experiences of their care. Factors contributing to suboptimal care were also identified. *Conclusions*. This way of thinking about how the fundamentals of care are experienced by patients may help to elucidate the complex processes involved around providing high quality fundamentals of care. This analysis illustrates the multiple dimensions at play. However, more systematic investigation is required with further refining and testing with wider healthcare user groups. The framework has potential to be used as a predictive, evaluative, and explanatory tool.

## 1. Introduction

Many healthcare systems face challenges related to the way they deliver fundamental aspects of patient care [1–5]. Typically, the literature on activities of daily living (ADL), self-care, and essentials or fundamentals of care presents them as discrete elements (such as elimination, mobility, dressing, comfort), assessed independently and pulled together in an overall assessment. With the rise of the patient/person-centred care (PCC) movement, more attention is being paid to patient involvement and participation, ways to enhance shared decision making and ensuring greater choice [6–8].

In addition to the patient-centred care literature, there is a growing need to provide a much more integrated health experience for both the patient and the practitioner [9, 10]. In particular Dossey [11] has built upon the seminal work of Florence Nightingale who argued that the role of the nurse is to place the patient in the best position for nature to heal him, taking account of the range of personal, physical, psychological and environmental conditions [11]. Dossey's Theory of Integral Nursing [9] starts with healing as the core ingredient and then builds up layers of integrated experience that optimises healing for both patient and nurse [9]. This work calls for full integration of experience. However, it is not clear from the literature how this integration actually happens in everyday encounters and in particular, how the fundamental aspects of care are addressed.

Nightingale and other nursing theorists were also the starting point for a program of work that has been led by a group of nurses called the International Learning Collaborative [12]. Taking a pragmatic approach, this group addressed the question of how to improve patients' experiences of the fundamentals of care in the acute hospital setting. It acknowledged the growing concern worldwide around standards of nursing care [13, 14] and hypothesised that one of the potential reasons for this was that nursing practice had abandoned the essential or core elements of its work. Kitson et al. (2010) undertook a narrative review of seminal texts (starting with Nightingale) and found that there was not consistency in the way the fundamentals of care were described in the literature [15] nor was there consistency in the underlying evidence base [16] or how nurses would learn how to practise those skills [12].

A subsequent line of investigation has focused on whether people who use health services (variously described as patients, clients, or survivors) have a view about the importance of the fundamentals of care and how they should be provided. In this work, Kitson et al. [17] found that when stroke survivors talked about physical elements of care (also called functional aspects), they talked about the psychosocial impact the experience had on them and then went on to describe the effect of the interaction with the healthcare professional (also termed transactional aspects). This link between the importance of functional quality (being able to undertake a task such as toileting a patient successfully) and transactional quality (being able to engage and connect with patients) has been identified by many researchers [5, 18, 19], particularly in relation to older peoples' experiences of acute hospital care [20, 21]. What is still not clear however, is how these aspects intersect at the point of an episode of basic care and what experiences at that moment determine whether it is more or less likely to be a positive or negative experience for the patient (and possibly for the carer as well).

In the Kitson et al. [15] narrative review, 14 core elements making up the fundamentals of care as conceptualized by nurses were identified (Table 1). These core elements are linked to what is commonly named as patient-centred care or person-centred care. The Fundamentals of Care Template [15] has subsequently been used as a tool to explore how different groups of patients describe the fundamentals of care and how they recounted their experiences of the acute phase of their care. The first group was stroke survivors [17] and the second group comprised people who have experienced cancer (breast, bowel, and prostate) [22].

These pieces of work have led to the development of the current conceptual framework which attempts to articulate the complexities involved in providing high quality, respectful fundamentals of care in the acute care setting. We argue that it could be a helpful framework to shape nurses' appreciation of the complexities involved in engaging patients in their activities of daily living or fundamentals of care. It also acknowledges the need for integration of the physical, psychosocial, and relational within each encounter. 

The research questions that shaped this study were:

1. Is it possible to develop a framework around patients' experiences of the fundamentals of care that explains and evaluates the quality of that encounter?
2. If this is the case, can the framework be tested using patient experiences of care as a first step to further validating?

## 2. Materials and Methods

A two stage process was undertaken. The first stage used the findings from the stroke [17] and other linked studies [12, 15, 16, 23] to create the conceptual framework. 

**Figure 1:**
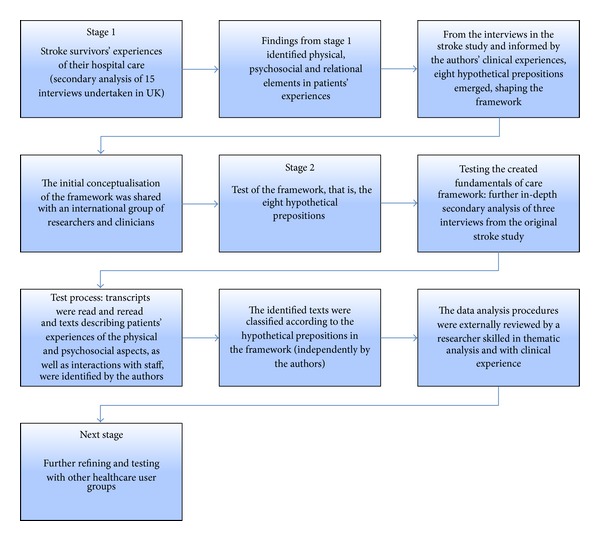
Processes and procedures in the development and testing of the conceptual framework.

 The second stage tested the hypothetical propositions emerging from the framework. This was done by undertaking a further in-depth secondary analysis of three purposefully selected interviews (from a total of 15) from the stroke survivor study [17]. Secondary analysis uses existing data, collected for prior purposes, in order to pursue a research interest which is distinct from the original work [24, 25].  Figure 1 describes the processes and the procedures in the development and testing of the conceptual framework.

The data used in the secondary analysis came from a re-analysis and interpretation of narrative interviews with stroke survivors from a study conducted in 2006-7, funded by a Big Lottery grant to the Managed Clinical Network on the Web (Scotland) and the Alliance for Self Care Research (University of Stirling) and updated with a further 11 interviews in 2011. The original study explored what it was like to live with a stroke including experiences of acute hospital care. Original interviews were conducted across the UK. Diverse purposive sampling was used in the original study to ensure variation in socio-demographic characteristics and types of experience. The stroke survivors (*n* = 15) differed in age (mid 30s to mid 80s) and gender (6 men and 9 women) and they were recruited from different sources. The interviews lasted for one to four hours and were audio-recorded and transcribed verbatim.

Ethical approval for use of the data for secondary analysis was obtained from the Multi-Centre Research Ethics Committee which approved the primary study. 

### 2.1. Data Analysis

Construction of the framework (the first stage) occurred following the analysis of the stroke survivor study [17]. Two members of this team (Alison L. Kitson, Åsa Muntlin Athlin) began to test a number of hypothetical situations and discussed how likely they would be in practice and whether they could explain or predict what patients had described. 

**Figure 2:**
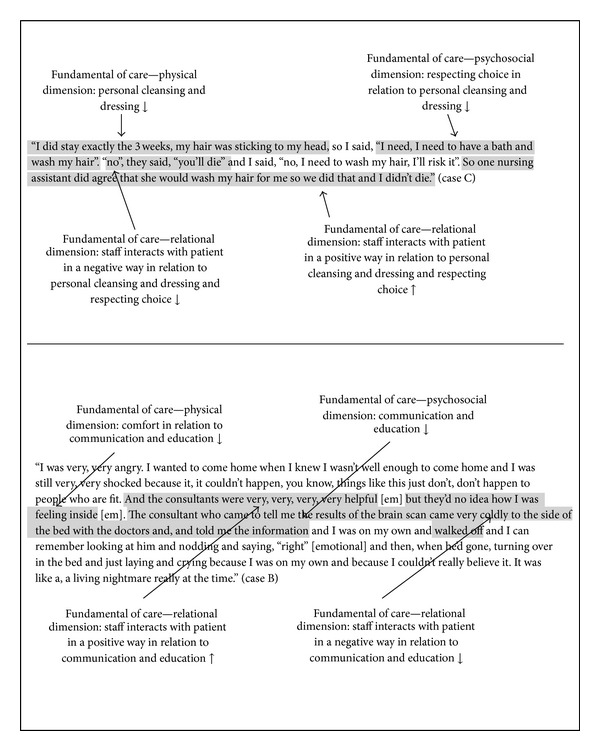
Data analysis process: examples of the text indicating the interpretation of the physical, the psychosocial and the relational dimensions.

Following the construction of the framework, three further stroke survivor cases were selected from the original stroke study [17]. Selected interviews included stroke survivorswith a moderate to high degree of impairment following strokeand the participants differed in gender and age. The narratives described both positive and negative experiences from the healthcare. Transcriptsoriginally coded by independently reviewers according to the Fundamentals of Care Template (Table 1) from the stroke study [17] were used. Each transcript was read and reread and texts describing care episodes of respondents' experiences of the physical and psychosocial aspects, as well as interactions with staff were recorded by Åsa Muntlin Athlin. Then each piece of identified text was reviewed and confirmed by Alison L. Kitson to ascertain whether it reflected not only the primary descriptor (the element of care which identified it in the first analysis as relating to elimination or personal cleansing and dressing or dignity for example) but also whether it described aspects of psychosocial and relational dimensions as hypothesised by the Fundamentals of Care Framework (see Figure 2 which describes the data analysis process). 

Following this first stage analysis, the second stage went on to test the hypothetical prepositions where each of the items of text as illustrated in Table 2 was classified according to the prepositions. This was done independently by the authors. 

These procedures were further externally reviewed by an independent researcher, skilled in thematic data analysis with clinical experience, who had not been involved in the original analysis of the stroke data. The number of care episodes describing interactions with members of staff for each case was counted and compared to the total number of quotations around a fundamental of care without reference to a staff member for each of the three cases. Types of health professionals described in the situations were identified and quantified. 

## 3. Results 

### 3.1. Stage 1: Developing the Framework

 Previous analysis of 15 stroke survivors' experiences indicated that there was a link between the way the physical task was undertaken (either by the person themselves or with help), the psychological impact that the physical task had on the person (depending on what happened and how it happened), and finally the way the interaction between the patient and the carer (nurse, allied health or doctor for example) was experienced and interpreted [17]. Thus for each interaction, using a framework that acknowledged the interplay of these three dimension, there would be at least eight possible categories that could describe the patient's experience of their care episode, ranging from a very positive experience where the physical care was good, the psychological experience was positive and the interaction between the carer and the patient was also considered to be positive to the less positive. At the other extreme, the framework would predict that there could be occasions where the patient would experience very poor physical care, deficient psychosocial support and a poor encounter with the carer (Figure 2, Table 2).

Turning the eight possible categories into propositions that could be tested, we then proceeded to structure the way we could test them. We hypothesised from the results of the stroke study [17] that the individual's experience of their *Fundamentals of Care (FOC)* is related to their need for support around the *physical dimensions of care (phy)*; their experience of the related *psychosocial elements of care (psy);* and their experience of the *relationship (rel)* with staff responsible for providing the fundamentals of care in a respectful, empathetic way. We also hypothesised that when individuals experience high quality physical, psychosocial, and interpersonal interactions with staff, that this would equate to significant elements of what is termed patient-centred care. However, it does not cover all the dimensions of patient-centred care (such as broader relational, co-ordination and systems dimensions) as described in the literature [18, 23]. Our focus was on the individual's fundamentals of care needs and how theses needs could be integrated within a patient/person-centred care philosophy. The following formula was used to summarise the core elements:

(1) $$\begin{array}{c} \text{Fundamentals}\;\text{of}\;\text{Care}=\int \left(\text{phy}\times \text{psy}\times \mathrm{rel}\right). \end{array}$$

In this framework fundamentals of care are considered to be a function of the follows.

**Figure 3:**
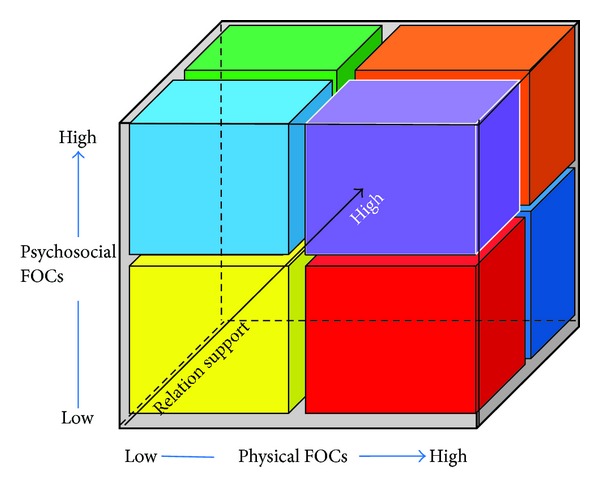
Fundamentals of Care: physical, psychosocial, and relational dimensions.

- The physical needs of the individual as articulated by them (or significant other) and assessed by the health professional and the way those physical needs are met: theoretically the experience of the physical care can range from Phy_low_ (Phy_↓_) to Phy_high_ (Phy_↑_). In Figure 3, the dimensions of *“FOC-Physical”* relate to activities such as mobility, elimination, eating and drinking, personal cleansing and dressing, comfort and pain control, safety, prevention and medication, rest and sleep, respiration, expressing sexuality, and temperature control. *“High”* in this situation means the inclusion of factors such as: the ability of the individual to set individual goals around each physical fundamental of care with relevant staff members and for those goals to be monitored in appropriate ways. *“Low”* is the failure to meet a person's physical needs in a timely and appropriate way and an absence of goal setting in any or all of these activities.
- The psychosocial needs of the individual as expressed by them (or significant other) and assessed by the healthcare professional and the way the attributes of the psychosocial needs are met: again this can theoretically range from poor experiences Psy_low_ (Psy_↓_) to positive experiences Psy_high_ (Psy_↑_). In Figure 3, the dimensions of *“FOC-Psychosocial”* relate to the experiences of dignity, respecting choice, privacy, and communication and education. *“High”* means the inclusion of factors such as: feeling involved, valued, and respected in the way fundamental needs are managed and met. *“Low”* is absence of the individual feeling or being involved or respected in any or all of the above factors.
- The interaction between the individual and the healthcare professional, termed, relational support (rel) as it is experienced can be low (little relational support or contact) or high (positive relationship established); Rel_low_ (Rel_↓_) to Rel_high_ (Rel_↑_). *“High”* means that the support provided by the staff member is respectful and covers both the physical aspects of care and the psychosocial elements. *“Low”* means that the staff member did not engage with the individual respectfully, appropriately, or empathetically in supporting them in the achievement of either physical or psychosocial aspects of care.

For example, a person's experience of their needs and how the staff interacted with them is based on, in Table 2, eight combinations of the three defining parameters. A “high” quality experience, where the individual's experience of a fundamental of care has been fully engaging and positive, is represented as, Phy_↑_ × Psy_↑_ × Rel_↑_. A “low” quality experience, where “minimum” engagement and empathy were experienced leading to poor physical care, poor psychosocial experience and poor relation with the staff member is represented as, Phy_↓_ × Psy_↓_ × Rel_↓_.

Individuals may experience poor physical care around eating and drinking, going to the toilet, and personal hygiene along side staff being friendly and kind to them. Such experiences may happen in clinical settings where physical care is not perceived as a core part of the registered nursing work role or where attention to technological or psychotherapeutic aspects are more important (Phy_↓_ × Psy_↑_ × Rel_↑_).

 Conversely, having a situation where individuals experience good physical care and where the professional and the individual have established a positive relationship, it would be difficult but theoretically possible to imagine such care failing to attend to the psychosocial wellbeing of the individual (Phy_↑_ × Psy_↓_ × Rel_↑_). Such situations may occur in clinical settings where there is a superficial attempt to engage patients in their psychological welfare and little emotional intelligence is displayed by staff, the relationship being centred on a superficial level of social niceties rather than any therapeutic underpinning. 

As can be seen from the examples above, some combinations are logically more congruent with a knowledge of practice; here Phy_↓_ × Psy_↓_ × Rel_↓_ and Phy_↑_ × Psy_↑_ × Rel_↑_ are hypothetically easier to populate with a patient narrative and context than some of the other categories which are theoretically possible but not as likely in practice. Having developed the framework with a level of face validity, we wished to proceed to the second stage and test the emerging framework against three purposefully selected cases. 

### 3.2. Stage 2: Preliminary Testing of the Dimensions Emerging from the Framework

#### 3.2.1. Characteristics of the Respondents

 Two of the cases were female and one male and their ages ranged from 40 to mid 70s. They all had experienced in hospital care and were interviewed originally in their own homes.

#### 3.2.2. Testing the Framework

Using transcripts coded according to the Fundamentals of Care Template, over 100 care episodes were identified between the three cases (36, 39, and 31, resp.). A total of 18 (case A), 25 (case B), and 26 (case C) care episodes described situations where staff were involved in the care (Table 3). Interactions with staff described by all three cases were found in each fundamental of care, besides: comfort, expressing sexuality, privacy, respiration, and temperature control. The majority of the interactions were to be found in communication and education, eating and drinking, and mobility.

The data from these cases confirmed that respondents reported a mix of poor and excellent experiences. Staff were connected to both poor and excellent experiences. All staff types (nurses, physicians, allied health professional, ancillary ward staff) were identified as contributing to the individual's experiences, both positively and negatively.

The reported experiences did consistently refer to physical, psychosocial and relational dimensions thereby confirming that these three elements are linked. However, as Table 3 shows over 60% of direct quotes around physical and psychosocial elements did explicitly refer to a staff member. For the remaining 35% of incidents we were unable to attribute the individual account to a particular staff member.

From the total of 106 reported experiences of fundamentals of care from three stroke survivors, it was possible to identify the extremes in the integrated Fundamentals of Care Framework, that is, Phy_↑_ × Psy_↑_ × Rel_↑_ and Phy_↓_ × Psy_↓_ × Rel_↓_, where the majority of experiences were allocated (Tables 4 and 5).

It was more difficult to identify pieces of text where respondents described excellent physical and psychological care without commenting on excellent relations with one or several staff members. This could infer that individuals are more likely to experience good care when they have what they perceive as a good relation with the carer and conversely a poor relationship is associated with more accounts of poor physical and psychosocial encounters (See Tables 4 and 5).

Another important observation was that the same respondent was found to recount incidents of both poor and excellent experiences for example, depending on the staff member and their own way of responding to the event and stage of recovery/level of dependency, for example,

* Interviewer: Tell me about your experience of, you know, having to have somebody help you with washing? *

*Case A: It was horrible because the particular nurse, the particular nurse that did it wasn't a very friendly nurse. She was very young and she did not chat. Some of the older ladies, as they're washing you, they'll chat and have a joke and a laugh and that's great and, and make comments and, but this particular, the very first time, I was mortified and I just couldn't do it and she said, “Well, I'll come and bath you” and she, she made an odd comment and she went away and I just cried. I just laid there and cried and I thought, “Nothing can be any worse than that”, to have somebody, because I'm aware of what's happening obviously (*Phys*_↓_*Psy*_↓_*Rel*_↓_ nurse). *

The above quote reflects the individual's distressing experience of having to wash themselves. This was contrasted with a further statement about how a negative experience with one staff member was transformed by interactions with another member of staff.

*Case A continued…andthen from then on, as I say, it, the next few times were easier and then gradually, they, they brought a, a thing of water to the bed and a flannel and some soap and the first day I washed myself in bed and I cleaned my teeth after about 4 days. It was absolutely wonderful and that was a turning point and then the next goal was I get into that bathroom and have a bath, well, I had a shower actually. It was much easier to sit in the shower at first but I managed it that way and there were grab rails at the side that you can hold on to and the, the nurses will stand by. There's no chance of you having a fall. They were great. They give you the respect. They'll stand outside the room while you get your underwear on or off or whatever. That, to me, was just the first stage in a long recovery but it was such an important stage being able to keep myself clean on my own. That sounds silly saying that. It's something you take for granted every day. You get up and have a wash but you haven't got the ability to clean your own teeth and illness is something that had never registered with me in my life before and I think this is probably why it came as such a shock (*Phy*_↑_*Psy*_↑_*Rel*_↑_ nurse).*

In the combination Phys_↑_ × Psy_↑_ × Rel_↑_, 24 accounts described interactions with allied health professionals compared to 15 accounts describing interactions with nurses and physicians. In the combination Phy_↓_ × Psy_↓_ × Rel_↓_, 26 accounts described interactions with nurses and physicians compared to none describing interactions with allied health professionals. This may be due to the fact that allied health professionals were able to work with individuals on targeted goal setting and establish a more one-to-one relationship, two factors which were found to be highly beneficial to stroke survivors during their recovery period [17]. 

## 4. Discussion

In addition to the conceptual development of the framework, this secondary analysis set out to answer whether the Fundamentals of Care Framework helped to categorise and explain incidents of positive and negative experiences of the fundamentals of care as experienced by stroke survivors. For each case studied there were examples of positive and negative experiences. For two cases there were more positive accounts (Case A and C) and for one (Case B), more negative accounts. While the theoretical dimensions of the framework identified eight potential combinations of experience (see Table 2) our analysis found that the majority of experiences were located in the extremes—either all positive (Phys_↑_ × Psy_↑_ × Rel_↑_) or all negative (Phys_↓_ × Psy_↓_ × Rel_↓_). This finding was as we had anticipated in that there were care situations hypothetically possible but some more likely than others to happen in practice (see column three in Table 2).

This would lead us to deduce that whilst theoretically possible the other permutations are less likely to be detected. However, another explanation may be that our data sources and our method of secondary analysis were not appropriate to glean this level of sensitive data and that we need to explore these dimensions in a different way. 

The data have demonstrated that an individual will recall a range of positive and negative experiences. The question arises as to when individuals make an overall assessment of their experience, how do they aggregate the multiple incidents into an overall experience of good or not so good care? Could we deduce from Cases A, B, and C which of these were most satisfied or had the most consistent care? Even though we do not know precisely how many staff these people interacted with, we know from the data that they were able to specifically identify those with whom they had positive experiences and those who caused them a level of distress.

The other issue this analysis raises is the multiplicity of staff engaged with any one person. How does the healthcare system ensure that there are certain methods of dealing with fundamental aspects of care that are acceptable and do not cause patient distress or anxiety? A document published by the NHS Institute for Innovation [5] also concluded that what matters to patients are the relational and functional aspects of care (page 11): “‘Relational' aspects of care (like dignity, empathy, emotional support) are very significant in terms of overall patient experience alongside ‘functional' aspects (access, waiting, food, noise)” [5].

Our analysis reinforces this but also points to the fact that for every physical act there is a linked psychosocial and relational impact. The integrated world of the individual is therefore challenged at every point in their care, from the simplest act of washing themselves to having to ask for help to go to the toilet or to eat. And if individuals are exposed to multiple staff in the course of their hospital stay there is more likelihood of relations not being forged in a meaningful or therapeutic way.

The analysis has demonstrated the complexity of delivering good care around the fundamentals. It has shown that respondents had variable experiences across the fundamentals and across staff groups (nurses, doctors, allied health, and ward ancillary staff) and within staff groups (different experiences of nurses). It also suggests that people do expect certain traits to be demonstrated in all staff, notably respect, keeping them involved and informed, and having a clear flow of information. These characteristics, while universally acknowledged in the patient-centred care literature [23], are still not routinely operationalised. By breaking activities down to their component parts may be one way of ensuring that the practical task (e.g., toileting) is always executed within a relationship that is respectful to the individual patient and which therefore is more likely to address the psychosocial dimensions of respect, dignity, involvement, and choice.

The question this poses then is how best to measure these integrated experiences? Foot and Cornwell [26] have described a number of ways that experiences of care are measured (e.g., experience, satisfaction, patient reported outcomes). These approaches record different experiences and are used by different groups in terms of feedback and action but few are able to provide feedback on the integrated patient experience. From the perspective of the Fundamentals of Care Framework, the question would be how a team of health professionals could utilise the framework to engage patients in giving them real time feedback on how they were caring for them [27]. If a patient knew that their interaction with a member of staff around going to the toilet would require competence in the physical execution of the task together with an attention to the patient's feeling of being respected and offered dignity and privacy, would this help improve experiences and would it enable both the carer and the patient to discuss the process? 

What is also important to note is the lack of contextual data to inform or explain the case responses. There is growing evidence around the impact poor staffing levels and low morale have on nurses' ability to provide high quality care [28] and it is emerging from this body of literature that in the absence of strategies to manage the constant and complex demands of patient care, nurses have to make decisions about how they ration their care [29]. The Fundamentals of Care Framework may be one approach to make explicit the emotional work necessary to provide basic physical care. 

Or perhaps the issue is not so much about relations but as Maben et al. [28] have found, it is more to do with the wider workforce context in which nursing care takes place. They found that patients were more likely to experience poor care when staff were overworked, had little support from co-workers or managers, had low levels of job satisfaction, and felt emotionally exhausted [28, 29]. This would lead one to ask whether the vision of patient-centred fundamentals of care based on effective relations between staff and patients is too idealistic in the current way that health systems are structured and maintained. Despite the many calls for more compassion [30] more dignified care [19], and more time to care [31] there are still many challenges.

One area not addressed in this research was any assessment of the clinical competence of the healthcare staff in their provision of the fundamentals of care. Respondents' narratives focused on their experiences and how they felt: there was no information about the appropriateness or clinical impact that the actions had on the recovery trajectory of patients. One would expect in the further refinement of the framework to be able to evaluate the clinical appropriateness of the action as well as the impact it had on the emotional wellbeing of the patient.

Different health professionals were involved in the interactions recounted by patients. Noteworthy are the positive accounts about the allied health professionals. This may be related to the structured way patients experience this therapy or it may also be related to how therapy is linked more to recovery and hope which in turn created a more positive recollection. Whatever the reason, it is important for nurses and doctors to reflect on how the more routine activities (e.g., washing, eating, going to the toilet, getting information about recovery, etc.) may need to be positioned in a more structured, therapeutic, goal setting framework so that individuals feel they are recovering. Equally, there may also be something about the time spent in a one-to-one relationship with the allied health professional. 

## 5. Limitations

This piece of work has several limitations and these should be taken into consideration when assessing the overall findings. Firstly, the data were derived from a subset of a secondary analysis conducted on a primary data set of narrative interviews of stroke survivors. Secondary analysis of data as a method has its own strengths and weaknesses. We conducted this analysis according to the methodological requirements of secondary analysis [24, 32, 33]. 

The three cases were deliberately selected for their rich content around their experiences. Therefore they could not be described as representative of either the whole group of stroke survivors or patients in general. Yet this was not the point of the analysis and we feel justified that by using data to test the coherence of the conceptual framework, we can now begin to test it in clinical settings with diverse patient groups.

As stated above, the client sample happened to be stroke survivors. We are not making any claims that the framework is more or less suited to this or any other group of patients. There may also be a case that by purposefully selecting three “rich” descriptive cases, we were skewing the results to fit into the proposed framework. We do not believe this to be the case as our findings do support consistent themes from the wider literature. However, we are further testing the eight positions within the framework. Other limitations of the primary data source are that it was collected in 2006-7 and is from one country (the UK).

Two other limitations could be the lack of validation of the experiences from a staff perspective and not being able to explore the respondent's understanding of the intersections between the functional (physical) and interactional (psychosocial and relational). In addition, the framework does not cover the wider contextual factors known to impact on nurses' ability to provide high quality nursing nor does the framework explore the elements of how the relationship between the nurse and the patient is established and maintained in the first place. These areas are part of an international collaboration which seeks to systematically explore these core aspects of care [12].

## 6. Conclusions 

The Fundamentals of Care Framework was found to classify three stroke survivors' experiences of care in a way that demonstrated the interplay of physical, psychosocial, and relational experiences. For care to be experienced positively, all three elements needed to be present in each encounter. Individuals recounted both positive and negative experiences of direct care, across professional groups and within groups. Respondents also reported more positive experiences related to some professional groups than others. This may be due to the time spent in a one-to-one relationship or the fact that encounters were more structured and goal oriented.

The framework for the purposes of this secondary analysis focused on individuals' in-hospital recollections of care. It did not assess the clinical appropriateness or quality of the care delivered nor was it able to cross validate individual experiences with staffs' perceptions. No contextual data were available to measure the impact of such factors as skill mix and staffing levels on quality of care. Further refinement of the framework will include staff and patient perceptions of discrete interactions in order to build up a more person-centred way of judging the quality of care. The Fundamentals of Care Framework is a way of integrating multiple interactions into an explanatory framework. It may also be useful as a predictive framework to indicate when care will not be integrated or person-centred. 

## Figures and Tables

**Table 1:** Fundamentals of care template (source Kitson et al., 2010 [15]).

Fundamental of care	Patient experience
Safety, prevention, and medication	
Communication and education	
Respiration	
Eating and drinking	
Elimination	
Personal cleansing and dressing	
Temperature control	
Rest and sleep	
Comfort (including pain management)	
Dignity	
Privacy	
Respecting choice	
Mobility	
Expressing sexuality	

**Table 2:** Eight hypothetical dimensions of the fundamental of care framework.

Dimensions of FOC	Individual experience	Likelihood in practice
Phy_↑_ × Psy_↑_ × Rel_↑_ (Orange in Figure 3)	Attention to physical, psychosocial needs and relational aspects in the majority of fundamentals of care	*Likely*—and aspirational. The ultimate target for patients and professionals and the goal of patient centred care

Phy_↑_ × Psy_↑_ × Rel_↓_ (Purple in Figure 3)	Attention to physical and psychosocial needs in the majority of fundamentals of care; little attention to relational aspects	*Likely*—in areas where more attempt is made to involve patients but staff have not been trained in terms of extending their interpersonal and relational skills or where they feel under time pressure to get the tasks done

Phy_↑_ × Psy_↓_ × Rel_↑_ (Blue in Figure 3)	Attention to physical and relational aspects in the majority of fundamental of care; little attention to psychosocial needs	*Unlikely*—possibly in settings where there is a superficial attempt to engage with patients but it is not authentic

Phy_↑_ × Psy_↓_ × Rel_↓_ (Red in Figure 3)	Attention to physical needs in the majority of fundamentals of care; little attention to psychosocial and relational aspects	*Likely*—in areas that have a biomedical approach to care and strict routines and where there are workforce shortages

Phy_↓_ × Psy_↓_ × Rel_↓_ (Yellow in Figure 3)	Majority of fundamentals of care characterised by lack of attention to physical and psychosocial needs and little empathy from staff	*Likely*—particularly in areas of low staffing, poor leadership, and low morale with high patient acuity

Phy_↓_ × Psy_↓_ × Rel_↑_ (Pink in Figure 3)	Lack of attention to physical and psychosocial needs but staff have demonstrated empathy and relationship building	*Unlikely*—but could happen in areas where there are skill and competency gaps and staff are well intentioned but inadequately prepared

Phy_↓_ × Psy_↑_ × Rel_↓_ (Turquoise in Figure 3)	Lack of attention to the majority of physical needs; more attention to psychosocial needs and little attention to the relational issues	*Unlikely*—may be seen in an environment that is required to implement a policy such as “dignity nurses” ref to improve patient experiences but not addressing physical needs

Phy_↓_ × Psy_↑_ × Rel_↑_ (Green in Figure 3)	Lack of attention to physical needs in the majority of fundamentals of care; good attention to psychosocial and relational aspects	*Less likely*—but could happen in some clinical areas where attention to physical aspects of care is a low priority and where patients' physical self care capacity was poor or inadequately assessed

**Table 3:** Number of care episodes describing interactions with staff identified in text coded according to the fundamentals of care template: three cases.

Fundamentals of care (FOC)	Case A *n* = 18 (out of *n* = 36)	Case B *n* = 25 (out of *n* = 39)	Case C *n* = 26 (out of *n* = 31)
Safety, prevention, and medication	1 (6)	2 (5)	1 (2)
Communication and education	2 (8)	13 (15)	6 (6)
Respiration	0 (0)	0 (0)	0 (1)
Eating and drinking	2 (4)	3 (7)	5 (7)
Elimination	3 (5)	2 (4)	2 (2)
Personal cleansing and dressing	1 (1)	2 (3)	3 (3)
Temperature control	0 (0)	0 (0)	0 (1)
Comfort (including pain management)	0 (1)	0 (0)	0 (0)
Dignity	0 (1)	1 (1)	6 (6)
Respecting choice	0 (0)	1 (1)	1 (1)
Mobility	8 (9)	1 (1)	2 (2)
Privacy	0 (0)	0 (0)	0 (0)
Rest and sleep	1 (1)	0 (2)	0 (0)
Expressing sexuality	0 (0)	0 (0)	0 (0)

Number in parentheses shows the total number of quotations for each fundamental of care, for each case.

**Table 4:** The fundamentals of care framework: individual descriptions of care episodes of more *positive* interactions with staff.

Fundamentals of care matrix	Number of healthcare professionals involved in the described interactions with the patient			Example of quotation
	Case A	Case B	Case C	
Phys_↑_ + Psy_↑_ + Rel_↑_ (*n* = 33)	7 OT 4 PT 2 Nurse 1 Staff	1 OT 1 PT 4 Nurse 4 Doctor	5 OT 4 PT 3 Nurse 1 Staff 2 ST	“One of the, the physios in the hospital had said, it, it's helpful to some people to do that because obviously at first you, I was told, “Don't be too hard on yourself and don't push yourself and wait until your body tells you that you can do it” but then rather than it all get on top of you and it'd be totally out of hand, be sensible and sit down and work out your limitations and what you can actually achieve without being totally wiped out and exhausted, so that you've still got something left afterwards… When you've done that thing, that is such a sense of achievement that you've done that and you then move on to the next and it makes you realise very, very small little steps get you a long way… and the sense of achievement is huge when, when you, you feel you haven't, you can't, you know, literally you can't get out of bed.” (Case B)

Phy_↓_ × Psy_↑_ × Rel_↑_	n/a	n/a	n/a	Not detected in cases

Phy_↓_ × Psy_↓_ × Rel_↑_ (*n* = 2)	1 Nurse	1 Doctor		“I was very, very angry. I wanted to come home when I knew I wasn't well enough to come home and I was still very, very shocked because it, it couldn't happen, you know, things like this just don't, don't happen to people who are fit. And the consultants were very, very, very, very helpful [em] but they'd no idea how I was feeling inside [em].” (Case B) “I did stay exactly the 3 weeks, my hair was sticking to my head, so I said, “I need, I need to have a bath and wash my hair”. “No”, they said, “You'll die” and I said, “No, I need to wash my hair, I'll risk it”. So one nursing assistant did agree that she would wash my hair for me so we did that and I didn't die.” (Case C)

Phys_↑_ + Psy_↓_ + Rel_↑_ (*n* = 1)	1 Nurse			“…if you're not used to asking people and you're used to being, being totally independent. I think that is the problem, you know. People are sometimes frightened to ask, you know, but nurses and doctors, nurses obviously in particular said, “We do this every day, you're not the first, we've, we've seen it and we do this every day. We've been doing it for years and we'll do it for years, so don't worry.” And once you can take that on board, once you can take that on board, you know [eh], that's a big hurdle to get over. As I said, you're, you're going to the toilet, you know, and they're actually cleaning you up, you know, and you're thinking to yourself, “God, this is terrible”, you know. But they're actually saying at the time, “Don't worry, this is, this is our job, we do this every day…” (Case A)

Staff: ward staff, nurse: registered nurse and nurse assistant, PT: physiotherapist, OT: occupational therapist, ST: speech therapist.

*n*: number of the combination for all three cases.

**Table 5:** The fundamentals of care framework: individual descriptions of care episodes of more *negative *interactions with staff.

Fundamentals of care matrix	Number of healthcare professionals involved in the described interactions with the patient			Example of quotation
	Case A	Case B	Case C	
Phys_↓_ × Psy_↓_ × Rel_↓_ (*n* = 23)	2 Nurse	6 Nurse 3 Staff 5 Doctor	3 Nurse 5 Staff 2 Doctor	“…because after the 3 weeks I hadn't been to the toilet at all and was desperate to go and said I needed to go to the toilet and they said, “You can't, if you go to the toilet, you'll die”. I said, “Well, I'm sorry, I just, don't you understand, I'm going to have to go. It's not a case of a choice, it's just I haven't been for 3 weeks and I need to go” and it was just going to happen. “I can't hold on to it any longer”. They said, “You can't go”. So I said I wanted to see the doctor because I have to be able to go to a toilet. …So I ended up going to see the on call doctor, who said, “No. If you go, you'll die” and then I asked to see the senior nurse to make a formal complaint and I did that. But in the end, they said, “No, you can't but you can have a pad on if you want to”. I said, “No”, I said, “You've taken everything from me, I've lost everything, I don't want to lose my dignity as well” so in the end they said, “You've got 2 choices, you can either have, have a pad on which is like a big nappy or we'll get the hoist, attach you to the hoist, lift you up over your bed and suspend you from 5 or 6 feet over your bed and you can just go to the toilet on your bed”. I said, “No, that's not acceptable. I'm not happy with that”. So in the end, I had the pad, which was pretty awful and, as I said like it took away the last of my dignity.” (Case C)

Phys_↑_ + Psy_↑_ + Rel_↓_ (*n* = 1)	1 Staff			“My right hand was worse than it had been last week. But once it was explained to me “Well you've been to the gym you've done a lot of walking you're tired and that's how it affects your stroke”. So you can take that on board, accept it and know you are going to be tired and know your hand's is going to be slow your right hand is going to be slow for a couple of days.” (Case A)

Phys_↓_ × Psy_↑_ × Rel_↓_ (*n* = 1)		1 Nurse		“I was given an antidepressant when I was in hospital by the doctors there but no assessment or anything by anyone from, from the mental health team.” (Case C)

Phys_↑_ × Psy_↓_ × Rel_↓_ (*n* = 8)	2 Nurse	1 Doctor	1 Nurse 4 Staff 1 Doctor	“…and sometimes I felt that the, the consultants didn't treat me as a person enough. It was more as if I were just another case or another piece of paper or another case study really, rather than me.” (Case B)

Staff: ward staff, Nurse: registered nurse and nurse assistant, PT: physiotherapist, OT: occupational therapist, ST: speech therapist.

*n*: number of the combination for all three cases.
